# The DEMATEL method explores the interdependent relationship structure and weights for diagnosis-related groups system

**DOI:** 10.3389/fpubh.2022.872434

**Published:** 2022-08-04

**Authors:** Tong Zou, Yanjun Jin, Yen-Ching Chuang, Ching-Wen Chien, Tao-Hsin Tung

**Affiliations:** ^1^Institute for Hospital Management, Tsing Hua University, Shenzhen, China; ^2^Taizhou Hospital of Zhejiang Province Affiliated to Wenzhou Medical University, Taizhou, China; ^3^Business College, Taizhou University, Taizhou, China; ^4^Institute of Public Health and Emergency Management, Taizhou University, Taizhou, China; ^5^Evidence-Based Medicine Center, Taizhou Hospital of Zhejiang Province Affiliated to Wenzhou Medical University, Linhai, China

**Keywords:** diagnosis-related groups (DRGs), the interdependent relationship structure and weights, decision-making trial and evaluation laboratory (DEMATEL), key factors, multiple criteria decision-making (MCDM)

## Abstract

**Purpose:**

This study constructs a structure of interaction between dimensions and criteria within the diagnosis-related groups (DRGs) system from a quantitative system and identifies key factors affecting the overall performance of medical services.

**Method:**

From September to December 2020, the influence relation structure diagram (IRSD) of the dimensions and corresponding criteria was developed from the practical experience of a group of domain experts, based on the DEMATEL method. Subsequently, all dimensions and criteria construct influential weights from a systems perspective. Finally, the main influential factors were identified based on the analysis results.

**Results:**

The IRSD results showed that, in the overall performance of medical services, “Medical service capacity (*C*_1_)” was the main influential dimension, influencing both “Medical service efficiency (*C*_2_)” and “Medical service safety (*C*_3_).” At the criteria level, “Case-mix index (CMI) (*C*_12_),” “Time efficiency index (C_21_),” and “Inpatient mortality of medium-to-low group (C_32_)” were the main influential criteria in the corresponding dimensions. The influential weight results showed that “Medical service capacity (*C*_1_)” was also a key dimension. “Case-mix index (CMI) (*C*_12_),” “Cost efficiency index (*C*_22_),” and “Inpatient mortality of medium-to-low group (*C*_32_)” were the key criteria in their respective dimensions.

**Conclusion:**

Patients and managers should first focus on the capacity of medical service providers when making a choice or deciding using the results of the DRGs system. Furthermore, they should pay more attention to medical safety even if it is not as weighted as medical efficiency.

## Introduction

Patients select the best medical service providers or managers based on their performance; the results of such scientific and reasonable evaluations assist them in making their decision. Some medical service performance evaluation tools have been proposed from various perspectives. For example, in the United States, the Joint Commission on Accreditation of Healthcare Organizations (JCAHO) implemented standardized, evidence-based performance measures in approximately 3,000 hospitals (accredited by the association) and compared the performance of these hospitals based on safety, patient satisfaction, efficiency, clinical quality, financial management, and medical expense indexes (1, 2). In Canada, hospitals in Ontario and Alberta, local governments, and the University of Toronto jointly use the Balanced Scorecard (BSC) as a tool to measure the medical performance of hospitals (3). BSC is further used in key process management and evaluates the vision and strategy of healthcare organizations through financial, customer service, internal business, and innovation dimensions (4).

Due to the impact of different case mix compositions, such as different characteristics and number of patients in each hospital (i.e., the type and number of cases admitted), a comparative analysis by the average length of stay, cost, or any other aggregate measure is not meaningful (5). Therefore, it is difficult for traditional tools, such as BSC and key performance indices (KPIs), to effectively solve this problem in the evaluation; thus, a reliable outcome cannot be guaranteed (i.e., based on the same evaluation perspective) (6). Therefore, objective and scientific evaluation of medical services is a complex and universal dilemma (7). To address this challenge, the usual strategy for the management of healthcare organizations is to introduce the concept of a “case-mix” (8),which can ensure the reliability of the medical services performance evaluation and systemic risk adjustment simultaneously (6, 9).

The most widely used risk-adjusting tool in the management of medical evaluations is the diagnosis-related groups (DRGs) system (10), which originated in the United States. The first generation of DRG systems was developed by Fetter et al. in 1976 (5). However, it was first applied to the hospital reimbursement system in New Jersey from 1978 to 1982 (11). Subsequently, it was chosen by the US Congress as a prospective payment system (PPS) for all MEDICARE patients (65-year-old and over) in 1983 (10). After its successful application in the United States, Europe, Australia, and some Asian countries, DRG systems have also been successively applied to manage medical services (10, 12).

In 2008, Peking University researchers completed the first edition of the Chinese mainland DRG system named PKU-DRGs, evaluating the performance of medical services in 21 large public general hospitals in Beijing (13). In 2009, China launched a major healthcare reform, with the DRGs' health insurance payment method as one of the important components (14). Since then, other parts of China, such as Henan Province and Shanghai, have introduced DRG systems to manage and research medical services (7, 15). In November 2021, the National Medical Security Administration of the People's Republic of China issued a document (16) on the need to reform DRGs payment methods within a three-year schedule from 2022 to 2024. This policy puts forward higher requirements for medical institution managers, requiring them to apply DRG systems to evaluate and improve the performance of medical services in hospitals.

The goal of the designed DRG system is to isolate problem areas such that corrective measures can be initiated (5). When using the DRG system to evaluate the performance of medical service providers, the dimensions and criteria are independent. For example, Jian et al. evaluated the performance of the medical services of 21 large public general hospitals in Beijing using the DRG system (13). The results show different dimensions and criteria for the different hospitals; therefore, it is impossible to provide a comprehensive evaluation and improvement direction. Or and Häkkinen conducted research on DRG systems in the United States and Europe (17). They found a relationship between capacity, efficiency, and safety, but failed to quantify and identify key factors. Feng et al. evaluated the medical service performance of five public hospitals in Jiading District, Shanghai from 2013 to 2019 (7). They found that the DRG-based inpatient service management (ISM) policy improved the capacity and efficiency of regional medical services. However, they did not explore the relationship between dimensions and criteria; therefore, they did not find the key factors and could not compare the overall performance of the medical services affected by the policy.

Therefore, from an academic perspective, the limitations of the DRG system should be improved, as their potential application has not been explored (5). This study applies the DEMATEL method to construct the influence relation structure diagram (IRSD) and influential weight within the DRG system. The IRSD can help decision-makers understand the interaction between all dimensions/criteria and identify their influence. The influential weights can help decision makers identify highly correlated factors within the DRG system. Based on the results of this study, hospital administrators and healthcare management consultants will be able to understand the operation of the DRG system in real-world environments from a systematic perspective.

The structure of this study is as follows: section Materials and methods describes the development background and index connotation of the DRG system, introduces the DEMATEL method and calculation steps, and describes the study designs. Section Results demonstrates the application of the DEMATEL method to obtain the IRSD and influential weights of the DRG system based on a group of experts' practical experience. Section Discussion analyses and discusses the results of the study. Section Conclusion presents the limitations and conclusions of this study.

## Materials and Methods

### The DRG system

The basic principle of the DRG system is to classify and group cases by disease type, treatment modality, and individual characteristics (18). Therefore, the DRG system is essentially a combination of cases (10). Ultimately, expectations of the output of medical services for cases in the same group are the same (5). Based on this advantage, the DRG system can solve the challenge of directly comparing medical services due to the diverse medical services being evaluated (13). Therefore, the DRG system can be used in payment, budget, and medical quality management for hospital management (19). The DRG system includes three dimensions and seven corresponding criteria; the details are listed in Table 1.

**Table 1:** The dimensions and corresponding criteria for DRG system.

**Dimension**	**Criteria**	**Description**
Medical service capacity (*C*_1_)	Number of DRG groups (*C*_11_)	The scope of conditions treated by different hospitals
	Case-mix index (CMI) (*C*_12_)	The standard measures for the severity of the patient's illness
	Total weight (*C*_13_)	The total output of hospital services
Medical service efficiency (*C*_2_)	Time efficiency index (*C*_21_)	The Time-efficiency of treatment for the same disease
	Cost efficiency index (*C*_22_)	The Cost-effectiveness of treatment for the same disease
Medical service safety (*C*_3_)	Inpatient mortality of low-risk group (*C*_31_)	Mortality rate of low-risk population in hospital
	Inpatient mortality of medium-to-low group (*C*_32_)	Mortality rate of medium-risk population in hospital

### The DEMATEL method

The Battelle Memorial Institute developed a systematic structural analysis method for assessing complex social network structure problems in the real world, namely the decision-making trial and evaluation laboratory (DEMATEL) method (20). Its main advantage is that it can analyze the interaction between subsystems in the system and visualize it using graph theory. Thus, the results can assist decision makers in focusing on a small number of core dimensions or criteria from an influence perspective (21). Researchers have applied this method to various topics such as medical decision-making (21, 22), nurse evaluation (23), education (24, 25), design evaluation (26), and open space planning (27). The calculation process is presented in our research and is implemented using Excel 2016 software (21–23). The specific steps are as follows:

Step 1: Establish an initialization influence relationship matrix ***T***.

Based on a set of 5-impact scales [e.g., (0) no impact to (3) extremely high impact], each domain expert fills in the degree of interaction between the dimensions/criteria. Subsequently, a direct influence matrix ***K*** = [_*k*_*ij*_]*n* × *n*_ is constructed. Then, the practical experience matrices of all experts' direct influence matrices are integrated into an initialization influence relationship matrix by the averaging method, as shown in Equation (1).


(1) $$T={\left[t_{ij}\right]}_{n\times n}={\left[\frac{1}{g}\sum_{u=1}^{g}k_{ij}^{u}\right]}_{n\times n},\;\;\;\;i,j\in \{1,2,\ldots ,n\}$$

where *g* is the number of domain experts and *n* is the number of criteria.

The consistency gap check for initializing the influence relationship matrix is shown in Equation (2).


(2) $$\begin{array}{c} \beta =\frac{1}{n(n-1)}\sum_{i=1}^{n}\sum_{j=1}^{n}\frac{\left|t_{ij}^{g}-t_{ij}^{g-1}\right|}{t_{ij}^{g}}\times 100\% \end{array}$$

where β is the confidence, and its value is <5% (the smaller the value, the smaller the average gap between the domain experts' practical experience, and the higher the stability of the results).

Step 2: Obtain a normalized influence relationship matrix ***N***.

An initialization influence relationship matrix ***T*** is converted into a normalized influence relationship matrix ***N*** using Equations (3, 4).


(3) $$\begin{array}{r} N=\frac{T}{\alpha } \end{array}$$

(4) $$\begin{array}{c} \alpha =\max\;\left\{\underset{i}{\max}\sum_{j=1}^{n}t_{ij},\underset{j}{\max}\sum_{i=1}^{n}t_{ij}\right\},\;i,j\in \{1,2,...,n\} \end{array}$$

Step 3: Derive the total influence relationship matrix ***H***.

The normalized influence relationship matrix **N** uses Equation (5) to calculate the result of multiple influences between the dimensions/criteria; then, the total influence relationship matrix ***H*** is obtained.


(5) $$\begin{array}{l} H=N+N^{2}+N^{3}+...+N^{\Omega }\\ \;=N{\left(I-N\right)}^{-1}\text{, when }\Omega \to \infty ,\;N^{\Omega }={\left[0\right]}_{n\times n} \end{array}$$

Step 4: Output four criteria and form an influence relation structure diagram.

The total influence relationship matrix ***H*** is obtained using Equations (6–9), which are four influence property indexes, namely, given influence, accepted influence, centrality, and cause degree for dimensions/criteria.


(6) $$\begin{array}{l} \text{Given influence}:\;s_{i}={\left[h_{i}\right]}_{n\times 1}\\ \;={\left[\sum_{j=1}^{n}h_{ij}\right]}_{n\times 1},\;i\in \{1,2,...,n\} \end{array}$$

(7) $$\begin{array}{l} \text{Accepted influence}:\;o_{i}={\left[h_{j}\right]}_{1\times n}^{{}^{'}}\\ \;={\left[\sum_{i=1}^{n}h_{ij}\right]}_{1\times n}^{{}^{'}},\;j\in \{1,2,...,n\} \end{array}$$

(8) $$\begin{array}{r} \text{Centrality}:\;s_{i}+o_{i} \end{array}$$

(9) $$\begin{array}{r} \text{Cause}-\text{effect}:\;s_{i}-o_{i} \end{array}$$

where ′ is the transposed symbol.

For centrality (*s*_*i*_+*o*_*i*_), a high value indicates a high degree of interaction with other dimensions/criteria; in contrast, a low value indicates a low degree of interaction. For cause-effect (*s*_*i*_−*o*_*i*_), a positive value (*s*_*i*_−*o*_*i*_>0) means that the main nature of the dimensions/criteria is influenced; in contrast, the main nature of the dimensions/criteria is to be affected (*s*_*i*_−*o*_*i*_ < 0). Finally, based on centrality (*s*_*i*_+*o*_*i*_) and cause-effect (*s*_*i*_−*o*_*i*_), an IRSD can be constructed, which shows the interaction of all dimensions or criteria in the real world.

Step 5: Build the influential weights.

For dimensions or criteria, centrality can be normalized to obtain the influential weight (i.e., the centrality in proportional form), as shown in Equation (10).


(10) $$\begin{array}{r} w_{i}=\frac{\left(s_{i}+o_{i}\right)}{\sum_{i=1}^{n}\left(s_{i}+o_{i}\right)} \end{array}$$

### Data collection and participant description

This study collected 19 DRG-related domain experts' practical experience (two clinical department directors, three clinicians, five hospital functional department staff, eight DRG management consulting analysts, and one government official). The proportion of male and female experts in this questionnaire survey was about the same, and the age was mainly distributed between 31 and 40 years, accounting for 58%. Most of the postgraduate students accounted for 53%. These domain experts are committed to providing objective and truthful data. The data survey was conducted between September and December 2020. The backgrounds of the 19 DRG-related domain experts are described in Table 2.

**Table 2:** The background and characteristics of DRG related domain-experts.

**Characteristics**	**Value (%)**
**Gender**
Male	9 (47%)
Female	10 (53%)
**Education**
Bachelor	4 (21%)
Master	10 (53%)
Ph.D.	5 (26%)
**Age**
Less than 30	4 (21%)
30–39	11 (58%)
40 and above	4 (21%)
**Department**
Company	7 (37%)
Clinic department	6 (32%)
Function department	5 (26%)
Government	1 (5%)
**Years of service**
Less than 2 years	12 (63%)
2–3	2 (11%)
3 and above	5 (26%)

## Results

### The results of interdependent structure

An initialization influence relationship matrix (***T***) (Table 3) developed from 19 DRG-related domain experts' perspectives was constructed using Equation (1). For the matrix (***T***), The statistical significance confidence and gap errors for the matrix (***T***) were 97.82% and 2.18%, respectively. Next, the matrix (***T***) used Equations (3–5) to derive the influence relationship matrix (***H***) (Table 4). Finally, the total influence relation matrix was transformed into four criteria (Table 5) and influential weights for the dimensions and criteria (Table 6) using Equations (6–10).

**Table 3:** Initialization influences relationship matrix **T**.

	***C*** **_11_**	***C*** **_12_**	***C*** **_13_**	***C*** **_21_**	***C*** **_22_**	***C*** **_31_**	***C*** **_32_**
*C*_11_	0.000	2.579	3.105	2.105	2.263	1.947	1.947
*C*_12_	2.737	0.000	3.316	2.737	3.000	2.579	2.526
*C*_13_	3.053	2.789	0.000	2.632	2.842	1.789	1.789
*C*_21_	2.000	2.368	2.632	0.000	2.895	1.895	1.842
*C*_22_	1.526	2.368	2.526	2.263	0.000	1.632	1.737
*C*_31_	1.632	2.316	1.842	1.895	2.053	0.000	1.842
*C*_32_	1.684	2.368	1.842	1.947	2.053	1.789	0.000

*The statistical significance confidence and gap errors were 97.82 and 2.18%*.

**Table 4:** Total influence relationship matrix **H**.

	***C*** **_11_**	***C*** **_12_**	***C*** **_13_**	***C*** **_21_**	***C*** **_22_**	***C*** **_31_**	***C*** **_32_**
*C*_11_	0.483	0.680	0.724	0.623	0.681	0.545	0.547
*C*_12_	0.704	0.641	0.829	0.739	0.809	0.649	0.649
*C*_13_	0.664	0.721	0.603	0.677	0.740	0.564	0.565
*C*_21_	0.574	0.655	0.687	0.498	0.694	0.530	0.529
*C*_22_	0.508	0.603	0.628	0.568	0.495	0.476	0.483
*C*_31_	0.493	0.580	0.575	0.531	0.581	0.372	0.472
*C*_32_	0.499	0.587	0.580	0.537	0.585	0.471	0.377

**Table 5:** The four criteria for dimensions and criteria.

	***r***_***i***_	***o***_***i***_	***r***_***i***_+***o***_***i***_	***r***_***i***_−***o***_***i***_		***r***_***i***_	***o***_***i***_	***r***_***i***_+***o***_***i***_	***r***_***i***_−***o***_***i***_
*C*_1_	1.970	1.834	3.804 (1)	0.136 (cause)	*C*_11_	4.283	3.925	8.208 (3)	0.358 (cause)
					*C*_12_	5.020	4.468	9.489 (1)	0.552 (cause)
					*C*_13_	4.533	4.626	9.159 (2)	−0.092 (effect)
*C*_2_	1.678	1.834	3.512 (2)	−0.156 (effect)	*C*_21_	4.169	4.173	8.342 (2)	−0.004 (effect)
					*C*_22_	3.762	4.585	8.347 (1)	−0.823 (effect)
*C*_3_	1.533	1.514	3.048 (3)	0.019 (cause)	*C*_31_	3.602	3.607	7.210 (2)	−0.005 (effect)
					*C*_32_	3.636	3.622	7.258 (1)	0.014 (cause)

*The value of () in centrality (r_i_+o_i_) is ranking; the value of () in cause-effect (r_i_−o_i_) is cause or effect group*.

**Table 6:** The influential weights for dimensions and criteria.

	**Local weight**	**Rank**		**Local weight**	**Rank**	**Global weight**	**Rank**
*C*_1_	0.367	1	*C*_11_	0.385	3	0.141	5
			*C*_12_	0.446	1	0.164	1
			*C*_13_	0.430	2	0.158	2
*C*_2_	0.339	2	*C*_21_	0.424	2	0.144	4
			*C*_22_	0.425	1	0.144	3
*C*_3_	0.294	3	*C*_31_	0.423	2	0.124	7
			*C*_32_	0.425	1	0.125	6

At the dimension level, the centrality results (*r*_*i*_+*o*_*i*_) show the degree of importance between the dimensions. “Medical service capacity (*C*_1_)” is the largest dimension of centrality (*r*_*i*_+*o*_*i*_), indicating that it is the most important dimension of the system. “Medical service efficiency (*C*_2_)” is secondary, and “Medical service safety (*C*_3_)” is the smallest. In the “Medical service capacity (*C*_1_)” dimension, “Case-mix index (CMI) (*C*_12_)” had the largest centrality, followed by “Total weight (*C*_13_)” and “Number of DRG groups (*C*_11_)” had the smallest centrality. In the “Medical service efficiency (*C*_2_)” dimension, “Cost efficiency index (*C*_22_)” is more central than “Time efficiency index (*C*_21_)”, indicating that more attention is needed in the “Medical service efficiency (*C*_2_)” dimension. In the “Medical service safety (*C*_3_)” dimension, “Inpatient mortality of medium-to-low group (*C*_32_)” is more central than “Inpatient mortality of low-risk group (*C*_31_)”, indicating that it requires more attention in the “Medical service safety (*C*_3_)” dimension.

The results of the cause-effect analysis (*r*_*i*_−*o*_*i*_) show the influencing properties of the dimension from the perspective of the net effect point of view. The causal effects of “Medical service capacity (*C*_1_)” and “Medical service safety (*C*_3_)” are both positive (*r*_*i*_−*o*_*i*_>0), which shows that both dimensions are cause-type dimensions, which influence more than they are affected. The causal effect of “Medical service efficiency (*C*_2_)” is negative (*r*_*i*_−*o*_*i*_ < 0), which shows that “Medical service efficiency (*C*_2_)” is a result-type dimension, which is more affected than its influences.

In the “Medical service capacity (*C*_1_)” dimension, the cause-effects of “Case-mix index (CMI) (*C*_12_)” and “Number of DRG groups (*C*_11_)” are greater than zero, which shows that both criteria are cause-type criteria, which influence more than they are affected. In addition, the cause-effect of “Total weight (*C*_13_)” is less than zero, which shows that “Total weight (*C*_13_)” is a result-type criterion that is affected more than the influence. In the “Medical service efficiency (*C*_2_)” dimension, the causal effects of “Time efficiency index (*C*_21_)” and “Cost efficiency index (*C*_22_)” are less than zero, which shows that both are result-type criteria. Meanwhile, “Cost efficiency index (*C*_22_)” has a lower causal effect than “Time efficiency index (*C*_21_),” indicating that “Time efficiency index (*C*_21_)” affects “Cost efficiency index (*C*_22_).” In the “Medical service safety (*C*_3_)” dimension, the causal effect of “Inpatient mortality of medium-to-low group (*C*_32)_” is greater than zero, which is a cause-type criterion. In addition, “Inpatient mortality of low-risk group (*C*_31_)” is less than zero, which is a result-type criterion. This indicates that “Inpatient mortality of medium-to-low group (*C*_32_)” affects “Inpatient mortality of low-risk group (*C*_31_).”

### The results of influential weights

At the dimension level, the weights range from large to small: “Medical service capacity (*C*_1_),” “Medical service efficiency (*C*_2_),” and “Medical service safety (*C*_3_),” respectively. In the “Medical service capacity (*C*_1_),” “Medical service efficiency (*C*_2_),” and “Medical service safety (*C*_3_)” dimensions, the weights are from largest to smallest: “Case-mix index (CMI) (*C*_12_),” “Total weight (*C*_13_),” and “Number of DRG groups (*C*_11_)”; “Cost efficiency index (*C*_22_)” and “Time efficiency index (*C*_21_)”; and “Inpatient mortality of medium-to-low group (*C*_32_)” and “Inpatient mortality of low-risk group (*C*_31_),” respectively. “Medical service capacity (*C*_1_)” has the largest weight at the dimension level. “Case-mix index (CMI) (*C*_12_),” “Cost efficiency index (*C*_22_),” and “Inpatient mortality of medium-to-low group (*C*_32_)” have the largest weights in their respective dimensions. This is mainly because the larger the centrality, the more important it is. Finally, the global weights of these seven criteria ranged from largest to smallest: “Case-mix index (CMI) (*C*_12_),” “Total weight (*C*_13_),” “Cost efficiency index (*C*_22_),” “Time efficiency index (*C*_21_),” “Number of DRG groups (*C*_11_),” “Inpatient mortality of medium-to-low group (*C*_32_),” and “Inpatient mortality of low-risk group (*C*_31_).”

## Discussion

### Research implications

This study constructs a structure of interaction between the dimensions and criteria within the DRGs system; it quantifies and identifies key factors affecting the overall performance of medical services. These results support those of previous studies. For example, Suarez et al. suggested that a professional neurocritical treatment and care team can significantly reduce in-hospital mortality and length of stay in a neuroscience critical care unit (28). In other words, improving medical capacity can reduce mortality and improve efficiency. Chowdhury et al. showed that medical capacity growth was driven mainly by improvements in technology rather than increases in efficiency, after analyzing panel data from Ontario hospitals from 2002 to 2006 (29). Barker et al. found that low-risk inpatients who were transferred from another hospital were three to six times more likely to die than those admitted from other sources; one important reason for such transfers was insufficient medical service capacity (30). In summary, this study proves the viewpoint of the above studies and provides a further quantitative description.

### Management practice based on IRSD

**Figure 1:**
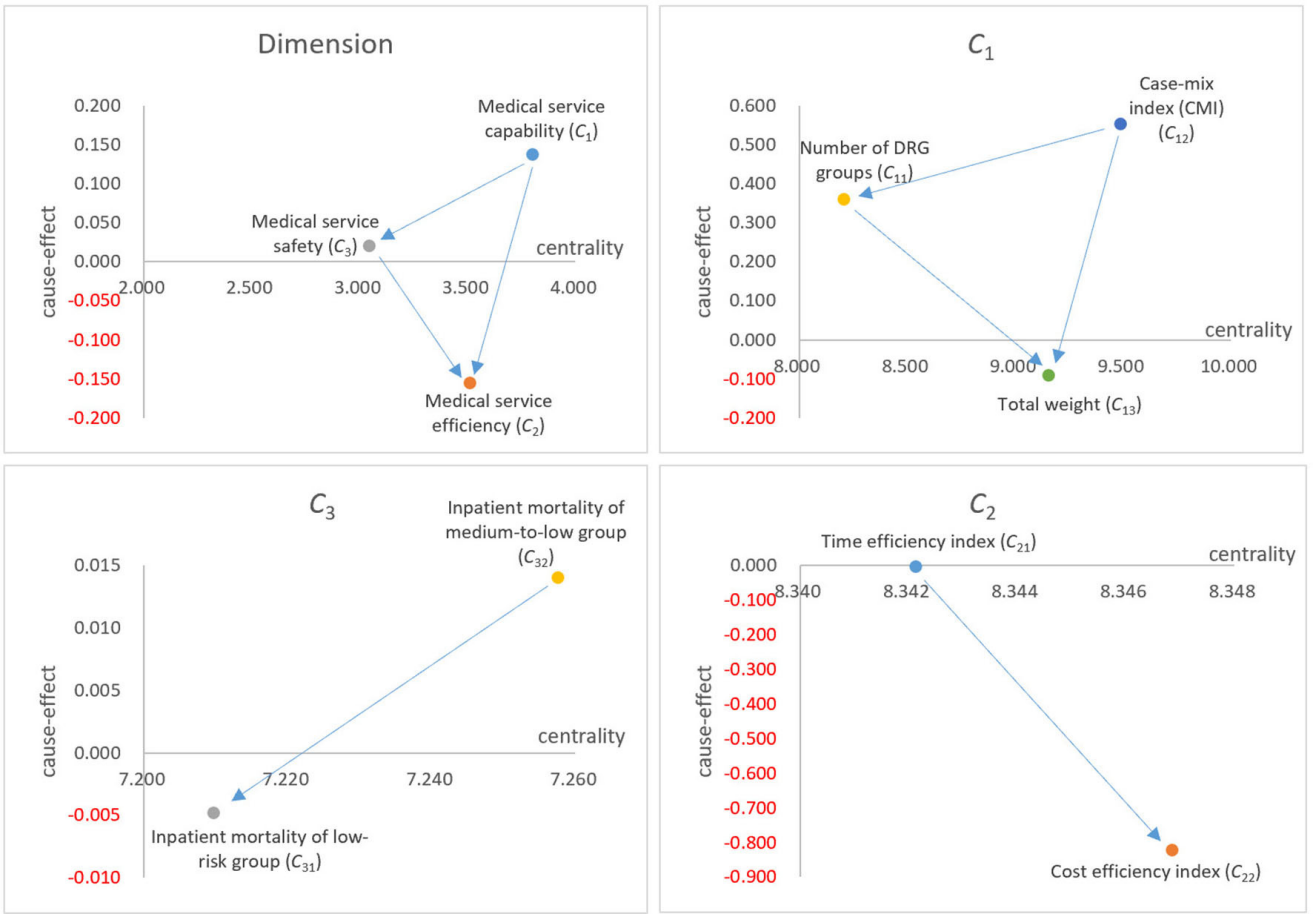
Influential relation structure diagram (IRSD) for this study.

The IRSD results showed an interdependent relationship between the dimensions and criteria of the DRG system, as shown in Figure 1. In the overall performance of medical services, “Medical service capacity (*C*_1_)” is the main influencing factor for improving “Medical service efficiency (*C*_2_)” and ensuring “Medical service safety (*C*_3_).” In clinical practice, improving medical service efficiency or ensuring safety should be based on a corresponding level of ability. Simultaneously, although “Medical service safety (*C*_3_)” has a lower centrality than “Medical service efficiency (*C*_2_),” it is a cause-type dimension, which affects “Medical service efficiency (*C*_2_)” while being affected by “Medical service capacity (*C*_1_).” This indicates that ensuring the safety of medical services is a top priority for medical institutions. “Medical service efficiency (*C*_2_)” is the result-type dimension affected by both “Medical service capacity (*C*_1_)” and “Medical service safety (*C*_3_).” In other words, improved efficiency must be based on satisfaction of capacity and safety elements.

In the “Medical service capacity (*C*_1_)” dimension, “Case-mix index (CMI) (*C*_12_)” was the main influencing factor, affecting both “Total weight (*C*_13_)” and “Number of DRG groups (*C*_11_).” In addition, there may be a positive correlation between “Case-mix index (CMI) (*C*_12_)” and “Medical service efficiency (*C*_2_)”. “Case-mix index (CMI) (*C*_12_)” reflects the technical difficulty level of treating cases, which may be the most important criterion in DRG systems. Although “Number of DRG groups (*C*_11_)” has a lower centrality than “Total weight (*C*_13_),” it is a cause-type criterion that affects “Total weight (*C*_13_)” while being affected by “Case-mix index (CMI) (*C*_12_).” It reflects the scope and breadth of disease treatment in medical institutions. “Total weight (*C*_13_)” is a result-type criterion affected by “Case-mix index (CMI) (*C*_12_)” and “Number of DRG groups (*C*_11_).” It reflects the total output of medical services, which is determined by the difficulty of treating cases and the breadth of diseases.

In the “Medical service efficiency (*C*_2_)” dimension, the centrality of “Cost efficiency index (*C*_22_)” is greater than that of “Time efficiency index (*C*_21_),” indicating that the utilization efficiency of medical expenses is more important. However, the cause-effect of “Time efficiency index (*C*_21_)” was greater than that of “Cost efficiency index (*C*_22_),” indicating that “Cost efficiency index (*C*_22_)” was affected by “Time efficiency index (*C*_21_).” In clinical settings, reasonable control of the number of days of hospitalization can effectively reduce the cost of hospitalization.

In the “Medical service safety (*C*_3_)” dimension, the centrality and cause-effect of “Inpatient mortality of medium-to-low group (*C*_32_)” are higher than those of “Inpatient mortality of low-risk group (*C*_31_),” indicating that “Inpatient mortality of medium-to-low group (*C*_32_)” is more important and has a greater impact on “Inpatient mortality of low-risk group (*C*_31_).” In clinical practice, there are higher standards and requirements to urge medical institutions to pay attention to and ensure the medical safety of patients.

Managers can use the results of this study to provide comprehensive performance evaluations and rankings to medical service providers, such as hospitals and clinical departments. The results of the IRSD can help managers find the direction of medical service improvement. In addition, the results of this study can help the DRG system become a timely and cost-effective management decision tool and retrospective monitoring system. First, the criteria were analyzed based on electronic data routinely collected in medical institutions. There was no need for additional resources or time. Through grouping and calculation of objective data, it can provide targeted warnings and reminders to hospital managers at any time and point out the direction of improvement based on the results of the IRSD.

### Academic and practical contributions

This study explored the relationship between the dimensions and criteria of the DRGs system from an academic perspective. This compensates for the deficiency of DRGs systems in medical service performance evaluation. In practical applications, this evaluation is based on the relationship between various dimensions and indicators and is closer to reality. Simultaneously, improvements in medical service performance will be targeted.

### Research and methodological limitations

First, the DRG system is a successful and useful system for health care delivery management worldwide. It has rapidly developed in China in recent years. However, it needs improvement to evaluate the performance of medical services in China in a more scientific, reasonable, and comprehensive manner. Second, the DRGs system is a multidisciplinary management system that involves a variety of disciplines, such as clinical medicine, statistics, and management. Experts from different professions may have different views on the DRG system, which will also lead to deviations from the results calculated through questionnaires and the real situation. Third, due to the complexity of medical services, we must further investigate whether the impact relationship obtained by the DEMATEL method is realistic.

### Future research directions

At present, the evaluation dimensions and criteria of DRG systems are not perfect, as they cannot accurately evaluate medical service performance. For example, Barker et al. (30) found that “inpatient mortality in the low-risk group” was invalid as a medical service safety criterion in some cases. In the future, the dimensions and criteria of DRG systems should be enriched and improved for more accurate evaluation. Simultaneously, researchers can construct a complete performance evaluation model based on the DRG system by using multi-attribute decision-making methods. Researchers can further use different versions of DRG systems to evaluate and compare the same hospital or clinical department and conduct a comparative study on the evaluation results. In addition, researchers can use methods, such as big data analysis and artificial intelligence, to predict medical service performance.

## Conclusions

In this study, DEMATEL was used to quantitatively determine the influence and weight of the various dimensions and criteria of the DRG system, which evaluates medical service performance. Managers can use the results of this study to provide comprehensive performance evaluations and rankings for medical service providers. The results have great potential to help DRGs become a timely and cost-effective management decision tool and retrospective monitoring system. In general, this study used DEMATEL to explore the influence relationship between various dimensions and criteria of the DRG system and to identify the key factors. Based on these results, the evaluation and improvement of medical service performance will be more targeted and practical.

## Data availability statement

The original contributions presented in the study are included in the article/supplementary material, further inquiries can be directed to the corresponding author.

## Author contributions

TZ and Y-CC conducted the study, drafted the manuscript, and calculated the results of this study and drew an influence relation structure diagram (IRSD). TZ participated in the design and data collection of the study. YJ participated in the design of the study and performed data analysis of the revised stage. T-HT and C-WC conceived the study and participated in its design and coordination. All of the authors read and approved the final manuscript.

## Conflict of interest

The authors declare that the research was conducted in the absence of any commercial or financial relationships that could be construed as a potential conflict of interest.

## Publisher's note

All claims expressed in this article are solely those of the authors and do not necessarily represent those of their affiliated organizations, or those of the publisher, the editors and the reviewers. Any product that may be evaluated in this article, or claim that may be made by its manufacturer, is not guaranteed or endorsed by the publisher.
